# Itaconate reduces viral endocytosis by targeting Cys128 of the adaptor-related protein complex 1 gamma 1 subunit in the host, providing a novel target for antiviral drug development

**DOI:** 10.1186/s43556-025-00348-6

**Published:** 2025-11-10

**Authors:** Xinqi Deng, Heng Chen, Zhixing Huang, Rongge He, Qinling Rao, Luni Xu, Zijian Xu, Naixuan Zhao, Yeqing Peng, Muxuan Li, Xi Liu, Tao Ma, Xiaolan Cui, Chunguo Wang

**Affiliations:** 1 Institute of Chinese Materia Medica, China Academy of Chinese Medical Sciences, Beijing, 100700 China; 2 Beijing University of Chinese Medicine, Beijing, 102488 China

**Keywords:** Itaconate, Post-translational modification, Virus, AP1G1, Clathrin

## Abstract

Traditional antiviral strategies primarily rely on vaccines and virus protein-targeting drugs, which adopt a virus-targeting approach. However, the rapid mutation of viruses often leads to vaccine failure and drug resistance, highlighting the limitations of these conventional methods. Consequently, the development of novel broad-spectrum, host-targeting antiviral strategies has become a major research focus. Itaconate, an endogenous immunomodulatory metabolite, inhibits viral replication via post-translational modifications; however, its mechanism in suppressing viral endocytosis remains unclear. This study demonstrates that itaconate inhibits viral endocytosis by covalently modifying the Cys128 site of the adaptor-related protein complex 1 gamma 1 subunit (AP1G1), thereby providing a new target for host-directed antiviral drug development. It was found that itaconate binds to AP1G1 at Cys128, impairing its interaction with clathrin, which inhibits clathrin-mediated viral particle uptake and reduces cellular susceptibility to infection (i.e., the likelihood of cells being infected by viruses and undergoing infection). Furthermore, the natural product Licochalcone B was identified as targeting the same site as itaconate. In both BEAS-2B cell models and mouse infection models, Licochalcone B reduced pulmonary viral loads by over 95%. This study is the first to propose and validate the feasibility of inhibiting broad-spectrum viral infection by targeting AP1G1, elucidating a novel molecular mechanism of itaconate-mediated regulation, offering a new target for broad-spectrum antiviral drug development, and identifying Licochalcone B as a promising broad-spectrum antiviral agent.

**Supplementary Information:**

The online version contains supplementary material available at 10.1186/s43556-025-00348-6.

## Introduction

Traditional antiviral methods adopt virus-targeting strategy and rely on vaccines and virus protein–targeting drugs [1–5]. However, the rapid mutation of viruses underscores the inherent limitations of these conventional antiviral approaches. Vaccines may prove ineffective against highly mutated viruses or in immunocompromised individuals, while frequent viral mutations can alter drug targets and the emergence of drug-resistant strains. In addition, many existing antiviral drugs exhibit off-target effects and may inadvertently disrupt host physiological functions, further limiting their clinical utility. These challenges highlight the urgent need for broad-spectrum antiviral strategies that are both effective and safe for clinical application.

Previous studies have demonstrated that endogenous itaconate regulates inflammatory immune responses and oxidative stress through transcriptional regulation [6–9]. Thus, itaconate plays a crucial regulatory role in viral infections, autoimmune diseases, sepsis, and disorders related to inflammation and oxidative stress induced by ischemia–reperfusion injury (IRI). However, derivatives of itaconate have been reported to reduce viral loads in the host during infectious diseases, and notably, does not depend on their anti-inflammatory properties [10]. This suggests that the antiviral effects of itaconate are not solely attributable to its classical immunomodulatory function. Instead, it appears to inhibit viral invasion during infection via a distinct and previously uncharacterized mechanism. Moreover, itaconate and its derivatives have demonstrated antiviral activity against a wide range of viruses, including SARS-CoV-2, Zika virus, and influenza virus, underscoring their broad-spectrum antiviral potential [11]. Among these derivatives, 4-octyl itaconate (4-OI) and dimethyl itaconate (DI) have shown particularly strong antiviral effects. For example, 4-OI inhibits SARS-CoV-2 replication in vitro and in ex vivo human airway epithelial cultures by activating the NRF2 pathway [12]. Additionally, 4-OI reduces Zika virus infection and inflammation in mouse models [13]. Collectively, these findings indicate that the antiviral mechanism of itaconate-independent of its traditional immunomodulatory activity-is broad-spectrum. Consequently, itaconate and its derivatives are promising candidates for future clinical development targeting diverse viral infections.

Host cell endocytosis is a critical link in virus invasion [14–18]. In recent years, host cell endocytosis has been identified as a promising regulatory target to restrict viral entry [19–22]. Adaptor protein (AP) complexes serve as the core regulatory complex of clathrin-mediated endocytosis [23, 24]. A variety of viruses, such as influenza virus and Ebola virus, utilize the AP complexes and the clathrin-mediated endocytosis pathway to enter cells [25]. In the AP complexes, inhibition of AP-1 complex activity can reduce viral invasion, thereby preventing early infection [26]. The AP-1 complexes are composed of four different subunits: γ, β1, μ1, and σ1. A genome-wide CRISPR screen identified the adaptor-related protein complex 1 gamma 1 subunit (AP1G1) as an essential host factor for the invasion of coronaviruses, including SARS-CoV-2 and MERS-CoV [27]. Consequently, AP complexes and AP1G1 have been confirmed to play crucial roles in viral endocytosis.

This study aims to develop novel broad-spectrum antiviral strategies, systematically elucidate the mechanisms by which itaconate exerts its broad-spectrum antiviral effects, and screen candidate drugs with broad-spectrum antiviral activity. Itaconate, containing an electrophilic α,β-unsaturated carboxylic acid moiety, can covalently modify cysteine residues of host defense-related proteins through Michael addition, thereby modulating host immune responses [11, 28, 29]. That is to say, itaconate can modulate protein function via cysteine modification. Based on this evidence, we hypothesize that a novel mechanism underlying the antiviral activity of itaconate involves the inhibition of viral invasion by targeting binding sites on cysteine residues in host proteins associated with endocytosis (e.g., AP1G1). This targeting modulates the function of these host proteins within the endocytosis pathway, offering a new strategy to minimize off-target toxicity while preserving broad-spectrum antiviral efficacy.

To validate this hypothesis, the study investigated the antiviral activity of itaconate using fluorescence microscopy and viral infection assays. The results demonstrated that itaconate significantly inhibits cellular endocytosis and reduces viral uptake. To identify the target protein through which itaconate exerts its antiviral effects, the study employed an itaconate-based clickable probe (ITAP) combined with click chemistry, pull-down assays, and lentivirus-mediated gene knock-down technology. This approach led to the identification and validation of AP1G1 as the key target protein interacting with itaconate. Further studies involving protein truncation, recombinant expression, and site-directed mutagenesis confirmed that itaconate binds to the Cys128 site of AP1G1. Molecular docking analysis revealed that the Cys128 site of AP1G1 is located within the interaction interface between AP1G1 and clathrin. Consequently, itaconate binding to the Cys128 site of AP1G1, thereby impairing the interaction between AP1G1 and clathrin, ultimately inhibiting viral entry. To advance the clinical application of AP1G1 Cys128, the study further screened small molecules from natural herbs using thermal proteome profiling (TPP). Licochalcone B was identified as an itaconate substitute, and its binding to Cys128 on AP1G1 was subsequently confirmed using limited proteolysis-mass spectrometry (LiP-MS). Furthermore, its antiviral efficacy was validated in BEAS-2B cells and mouse models. This study provides an approach for resisting broad-spectrum virus invasion and identifies a relevant prodrug molecule for further development.

## Results

### Itaconate covalently modifies host protein cysteines, reducing host cell susceptibility to viral infection

**Fig. 1:**
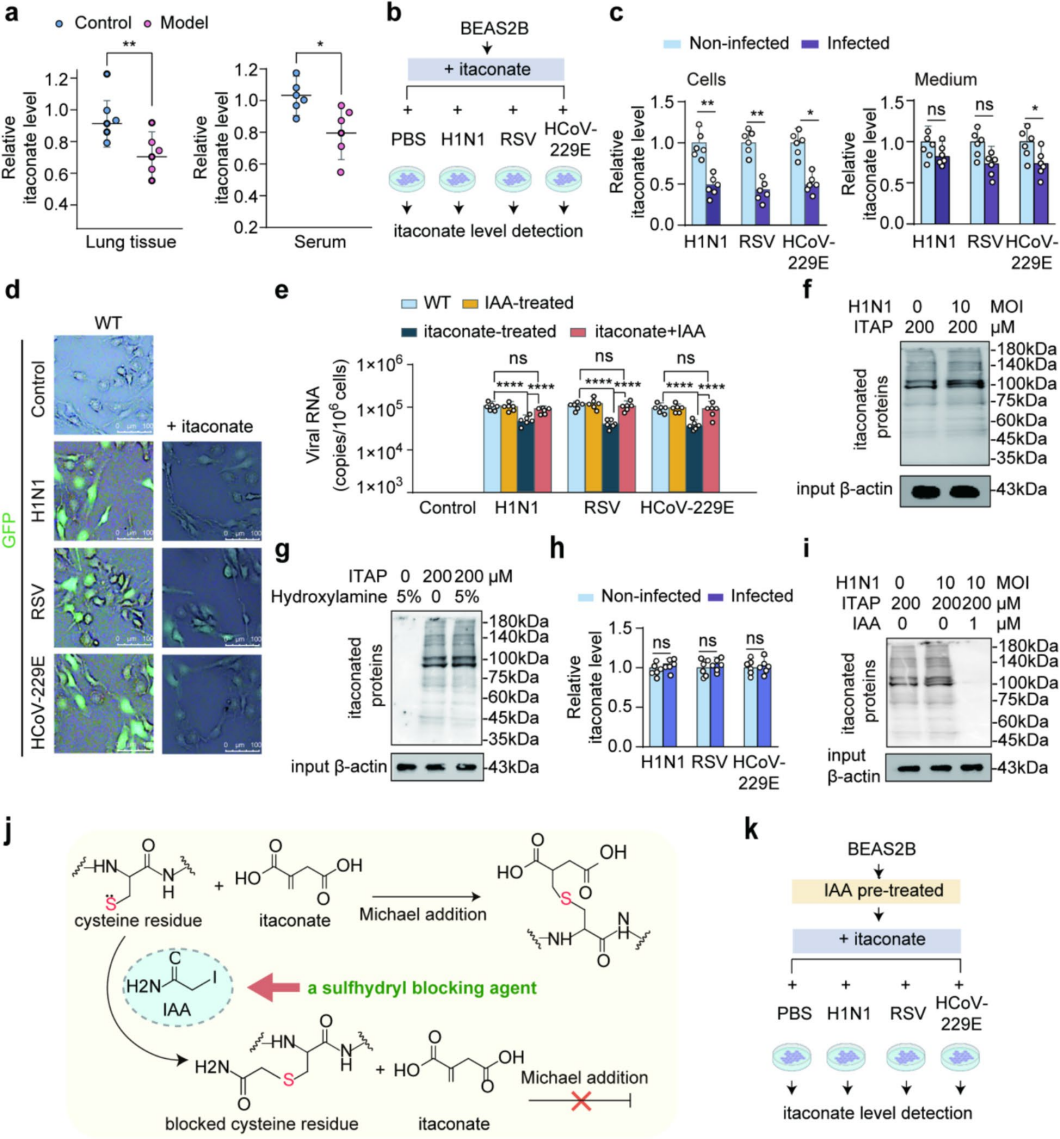
**a** The level of itaconate in mice lung tissue and serum upon viral infection. Male specific pathogen-free Kunming (KM) mice in the model group were intranasally inoculated with H1N1 (100 TCID_50_ in 40 μL PBS) once daily for 5 consecutive days; control mice received PBS only, *n* = 6, *p < 0.05, **p < 0.01. **b** BEAS-2B cells were infected with H1N1, RSV, and HCoV-229E at MOI = 2 for 24 h. **c** The level of itaconate in BEAS-2B cells and corresponding culture supernatants, *n* = 6, *p < 0.05, **p < 0.01. **d** BEAS-2B cells were infected with GFP-tagged viruses of H1N1, RSV, and HCoV-229E (MOI = 2, 24 hpi). The GFP signal was attenuated in the itaconate administration group compared with the WT group, indicating reduced virus invasion, *n* = 3. Scale bar = 100 μm. **e** Viral RNA level in infected cells. Cells were treated with itaconate, IAA, and itaconate + IAA. In the itaconate + IAA group, cells were pretreated with IAA (1 μM) for 24 h, and then subjected to itaconate administration (200 μM), *n* = 6, ****p < 0.0001. **f** Western blot analysis of protein itaconation in BEAS-2B cells infected with H1N1 influenza virus (MOI = 0 or 10) for 24 h (indicated with ITAP, 200 μM). Itaconation signal was detected by biotin-click assay. β-actin of input served as a loading control. **g** Hydroxylamine treatment did not remove itaconation signal of proteins, indicating that itaconate binds to proteins with thioester covalent bond. Itaconation signal was detected by biotin-click assay. β-actin of input was used as a loading control, *n* = 3. **h**, **i** Itaconation in cells was inhibited with IAA pretreatment. The level of itaconate in BEAS-2B cells pretreated with IAA (1 μM). No change in itaconate level of cells that exposed to virus infections of H1N1, RSV, and HCoV-229E (MOI = 10), *n* = 6 (**h**). Changes in bound itaconate content after IAA treatment. Western blot analysis of protein itaconation in BEAS-2B cells under three conditions: (1) H1N1 MOI = 0, ITAP 200 µM, IAA 0 µM; (2) H1N1 MOI = 10, ITAP 200 µM, IAA 0 µM; (3) H1N1 MOI = 10, ITAP 200 µM, IAA 1 µM, 24 h. Itaconation signal was detected by biotin-click assay, β-actin of input served as a loading control, *n* = 3 (**i**). **j**, **k** Scheme of blocking thioester covalent bond of itaconate in BEAS-2B cells with IAA. The α,β-unsaturated carboxylic acid group in itaconate and its Michael addition. The carbon–carbon double bond in α,β-unsaturated carboxylic acid can undergo a Michael addition reaction with the exposed sulfhydryl group on cysteine to form a strong covalent bond. IAA can block sulfhydryl groups, preventing them from undergoing Michael addition reactions with itaconate (**j**). After IAA pretreatment, cells were cultured in itaconate supplement medium and exposed to virus infections of H1N1, RSV, and HCoV-229E (MOI = 10) (**k**)

We found that 5 days after infection with a 100 TCID50 dose of influenza A virus (H1N1), the levels of itaconate were decreased in the lung tissue and serum of the model group mice compared to the uninfected control group (Fig. 1a). To determine whether this alteration in itaconate levels occurs in other viral infections, respiratory syncytial virus (RSV) and human coronavirus 229E (HCoV-229E) were also used to infect BEAS-2B cells, and intracellular itaconate concentrations were measured (Fig. 1b). The concentrations of itaconate in both BEAS-2B cell body and the culture medium showed a decrease upon virus infections. Notably, the decline in itaconate levels was more pronounced within the cells compared to the culture medium (Fig. 1c). This suggests that itaconate is consumed in host cells during viral infections. Using GFP-labeled viruses, viral entry was tracked, and the intracellular fluorescence intensity of H1N1, RSV, and HCoV-229E was markedly reduced in itaconate-treated cells (Fig. 1d). This finding suggests a potential association between the consumption of itaconate and the susceptibility of cells to virus.

It was found that the susceptibility of cells to the virus was significantly reduced following itaconate treatment (Fig. 1e). To investigate the modification of host proteins by itaconate during viral infection, an itaconate-based clickable probe (ITAP, Fig. S1) was used, revealing an increase in itaconate-protein adducts (Fig. 1f). This covalent binding was confirmed by its resistance to hydroxylamine cleavage (Fig. 1g), consistent with previous reports [30]. Furthermore, treatment with the sulfhydryl-blocking agent iodoacetic acid (IAA) maintained itaconate levels in the culture medium and markedly weakened the ITAP labeling signal (Fig. 1h, i). The significant inhibition of itaconation upon IAA treatment (Fig. 1j, k) suggests that itaconate binds to cysteine residues on host proteins. Consistently, the reduced susceptibility to viral infection caused by itaconate treatment was restored when cells were pretreated with IAA (Fig. 1e). Together, these results suggest that itaconate likely modifies cysteine residues on host proteins, which may contribute to the susceptibilities of cells to virus.

### Itaconate reduces host susceptibility to viruses by targeting AP1G1

**Fig. 2:**
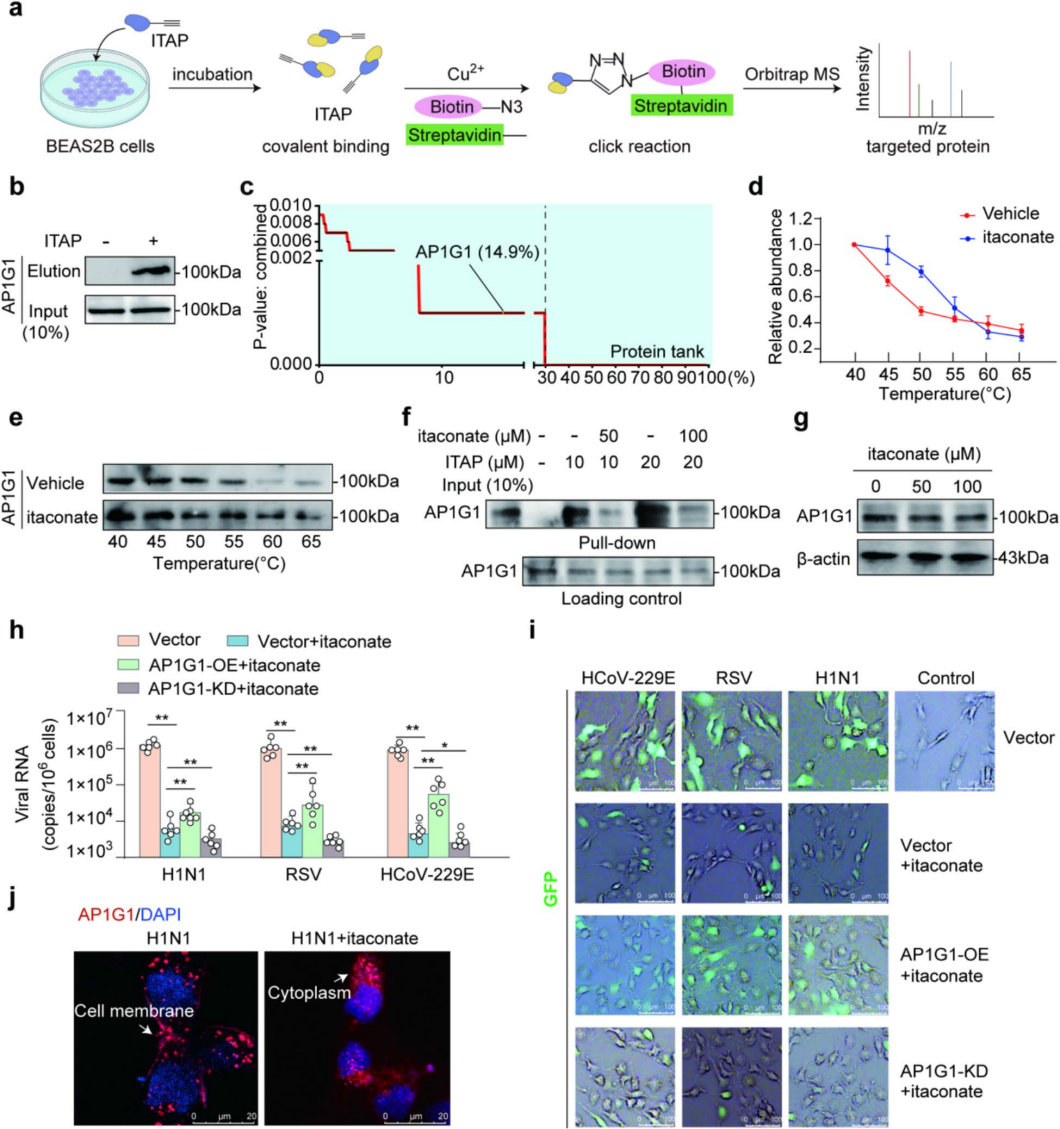
**a** Workflow of labeling and identifying the itaconate-targeted proteins with CuAAC-mediated click chemical reaction and Orbitrap MS. **b** ITAP pull-down assay indicates the interaction between itaconate and AP1G1 recombinant protein, *n* = 3. **c** Using a CuAAC-based bioorthogonal chemistry assay, ITAP was employed to enrich itaconate-modified target proteins. AP1G1 was identified as a high-ranking candidate, ranking in the top 14.9% among potential itaconate-targeted proteins. **d**, **e** Itaconate treatment significantly altered the thermal stability of AP1G1. Thermal shift curve of AP1G1 was determined by Cellular Thermal Shift Assay (CETSA)-Western blot at 40, 45, 50, 55, 60, and 65 ℃. Western blot image (**e**) and quantitative analysis (**d**) showing increased thermal stability of AP1G1 in the itaconate-treated group compared to the vehicle, *n* = 3. **f** Itaconate competitively binding on AP1G1 and impede the pull-down efficacy of ITAP. BEAS-2B cell lysates were incubated with ITAP (10 μM or 20 μM) upon the competition of itaconate (50 μM or 100 μM) under native conditions. After incubation, ITAP-bound proteins were captured with biotin-click and streptavidin-conjugated beads, *n* = 3. **g** AP1G1 expression in BEAS-2B cells after the administration of itaconate at concentrations of 0, 50, 100 μM. Itaconate administration does not alter the expression of AP1G1, *n* = 3. **h**, **i** AP1G1 serves as a key target of itaconate in inhibiting virus invasion. Viral RNA levels in BEAS-2B cells that were infected with H1N1, RSV, or HCoV-229E viruses (MOI = 2, 24 hpi) under four conditions: vector control, vector + itaconate, AP1G1 over-expression + itaconate, and AP1G1 knock-down + itaconate. Viral RNA copies were quantified by qPCR, *n* = 6, *p < 0.05, **p < 0.01 (**h**). BEAS-2B cells were infected with GFP-tagged viruses of H1N1, RSV, and HCoV-229E (MOI = 2, 24 hpi). The GFP signal was attenuated in the sh-AP1G1 group compared with the WT group, indicating reduced virus invasion, *n* = 3. Scale bar = 100 μm (**i**). **j** Immunofluorescence assay showing the subcellular localization of AP1G1 following itaconate treatment. Itaconate administration altered the subcellular distribution of AP1G1, *n* = 3. Scale bar = 20 μm

To identify the target proteins of itaconate, we employed ITAP in the copper(I)-catalyzed alkyne-azide cycloaddition (CuAAC) chemical bioorthogonal experiment (Fig. 2a). Among the ITAP-pulled down proteins, AP1G1 ranked in the top 14.9% (Fig. 2b, c). In addition to its role as a SARS-CoV-2 host dependency factor [27], AP1G1 functions as a subunit of the AP-1 clathrin adaptor complex, which mediates protein sorting between the trans-Golgi network and endosomes [31, 32]. This adaptor complex recruits clathrin to facilitate vesicle formation, a process hijacked by various viruses during entry and intracellular trafficking. Furthermore, AP1G1 exhibited significantly altered thermal stability following itaconate treatment (Fig. 2d, e). Excess itaconate can also competitively bind to ITAP' s AP1G1-binding site in situ (Fig. 2f). These results further validate that AP1G1 is a target protein of itaconate.

To investigate the functional regulatory mechanism of itaconate, we examined the effects of itaconate treatment on AP1G1 expression levels, as well as the impact of AP1G1 over-expression and knock-down on itaconate' s antiviral activity. The results showed that administering itaconate did not affect AP1G1 expression levels (Fig. 2g). Therefore, itaconate does not appear to influence AP1G1 function by altering its expression. Overexpression of AP1G1 weakened the antiviral effect of itaconate (Fig. 2h, i). Conversely, knock-down of AP1G1 (Fig. S2a) in BEAS-2B cells enhanced their resistance to viral invasion by H1N1, RSV, and HCoV-229E (Fig. 2h, i). These findings suggest that itaconate may reduce host susceptibility to viruses by targeting AP1G1.

### Itaconate interferes with the interaction between AP1G1 and clathrin

AP complexes can facilitate viral invasion through clathrin-mediated endocytosis [23]. It was observed that AP1G1 interacts with clathrin during infection by H1N1, RSV, and HCoV-229E, but not under uninfected conditions. This suggests that AP1G1, similar like AP complexes, may facilitate viral endocytosis through its interaction with clathrin (Fig. S2b, c). Immunofluorescence and western blot analyses showed that itaconate treatment retained AP1G1 in the cytoplasm, preventing its translocation to the cell membrane (Figs. 2j, S2d).

**Fig. 3:**
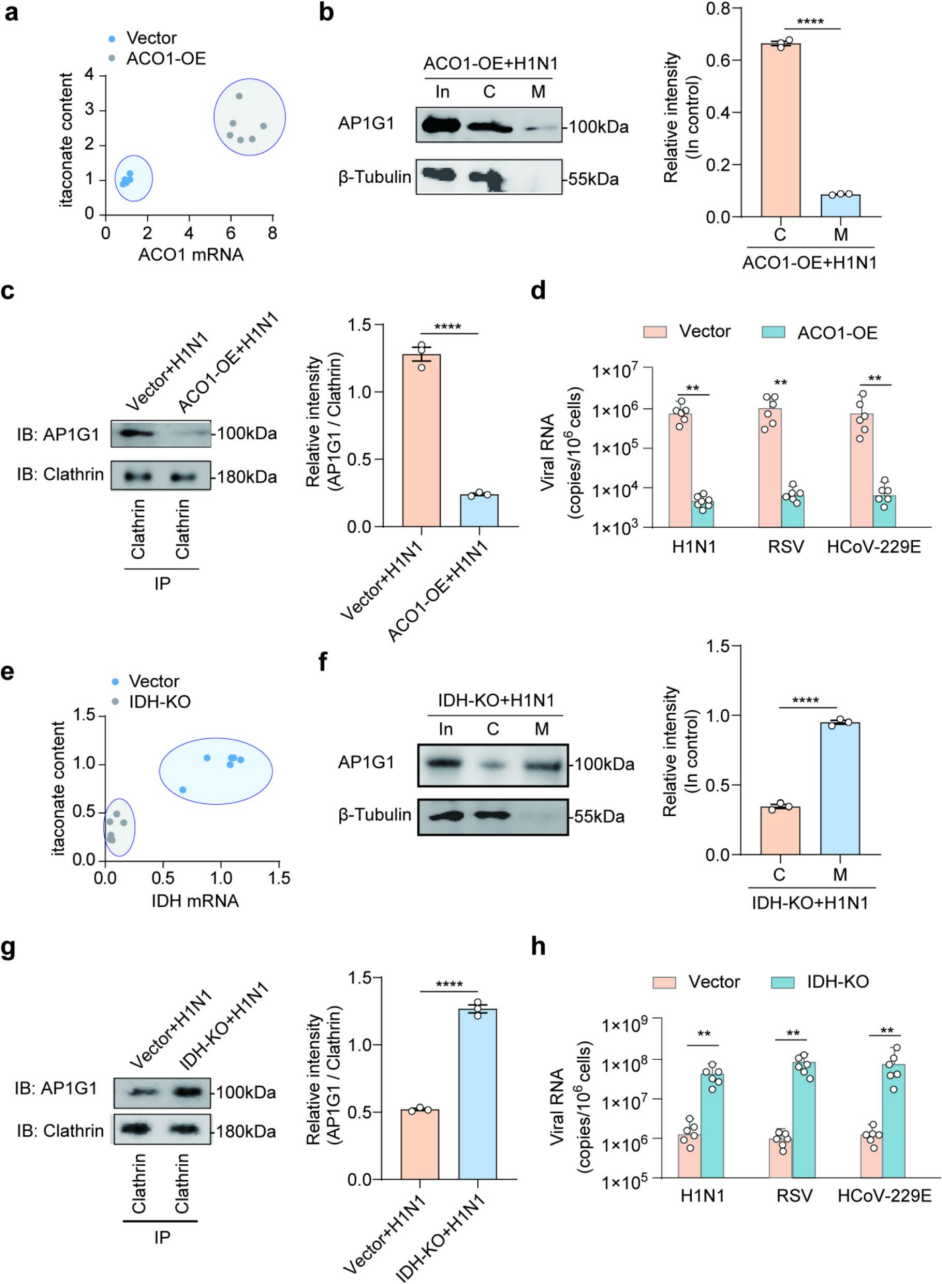
**a** Enhancement of endogenous itaconate expression through over-expressing ACO1, *n* = 6. **b** Western blot analysis showing subcellular localization of AP1G1 during H1N1 virus infection following ACO1 over-expressinon. In = input, C = cytoplasm, M = cell membrane. The relative expression levels (input control) were quantified in the right panel, *n* = 3, ****p < 0.0001. **c** CO-IP experiments show that the interactions between AP1G1 and clathrin are significantly diminished following ACO1 over-expression during H1N1 virus infection. The relative expression levels (clathrin control) were quantified in the right panel, *n* = 3, ****p < 0.0001. **d** Viral RNA levels in vector and ACO1 over-expression BEAS-2B cells. Cells were infected with H1N1, RSV, and HCoV-229E viruses (MOI = 2), *n* = 6, **p < 0.01. **e** Reduction of endogenous itaconate expression through IDH knock-out, *n* = 6. **f** Western blot analysis showing subcellular localization of AP1G1 during H1N1 virus infection following IDH knock-out. The relative expression levels (input control) were quantified in the right panel, *n* = 3, ****p < 0.0001. **g** CO-IP experiments show that the interactions between AP1G1 and clathrin are significantly enhanced following IDH knock-out during H1N1 virus infection. The relative expression levels (clathrin control) were quantified in the right panel, *n* = 3, ****p < 0.0001. **h** Viral RNA levels in vector and IDH knock-out BEAS-2B cells. Cells were infected with H1N1, RSV, and HCoV-229E viruses (MOI = 2), *n* = 6, **p < 0.01

To investigate the role of itaconate in regulating the subcellular localization of AP1G1 and its impact on AP1G1-clathrin interactions, endogenous itaconate levels were increased by overexpressing aconitase 1 (ACO1) (Fig. 3a). ACO1 is a mitochondrial enzyme that catalyzes the decarboxylation of cis-aconitate, an intermediate metabolite in the tricarboxylic acid (TCA) cycle, to produce itaconate. Over-expression of ACO1 enhances its catalytic activity, thereby increasing itaconate production. Under H1N1 infection, increased endogenous itaconate resulted in greater cytoplasmic retention of AP1G1 and reduced membrane localization (Fig. 3b). Concurrently, elevated endogenous itaconate markedly inhibited the interaction between AP1G1 and clathrin (Fig. 3c). These findings suggest that itaconate regulates AP1G1 subcellular localization and modulates its interaction with clathrin under viral infection conditions.

The impact of itaconate-mediated regulation of AP1G1 and clathrin on viral invasion was further examined in three viral infections: H1N1, RSV, and HCoV-229E. Compared with controls, elevated endogenous itaconate significantly reduced viral RNA levels and effectively inhibited viral entry (Fig. 3d). These results suggest that itaconate’ s regulation of AP1G1 subcellular localization and its interaction with clathrin may influence the cellular endocytosis of viruses.

To further validate these findings, endogenous itaconate levels were reduced through reverse modulation. Isocitrate dehydrogenase (IDH) participates in the TCA cycle and influences itaconate metabolism [33]. Itaconate, in turn, can inhibit IDH activity via negative feedback regulation [34]. Therefore, IDH knock-out may affect itaconate production. IDH knock-out drastically reduced itaconate levels (Fig. 3e). This effect likely occurs because both α-ketoglutarate (α-KG), produced by IDH, and itaconate competitively inhibit TCA cycle-associated TET family dioxygenases (mainly TET2) [35]. Following IDH knock-out, reduced α-KG levels may trigger compensatory downregulation of itaconate to maintain TCA cycle homeostasis. Consistent with expectations, depletion of endogenous itaconate increased AP1G1 localization at the cell membrane and decreased its cytoplasmic distribution (Fig. 3f). Additionally, AP1G1 exhibited significantly enhanced interactions with clathrin (Fig. 3g). Under H1N1, RSV, and HCoV-229E infections, depletion of endogenous itaconate significantly increased viral RNA levels (Fig. 3h).

### Itaconate binds to Cys128 of AP1G1, impairing AP1G1-clathrin interaction

**Fig. 4:**
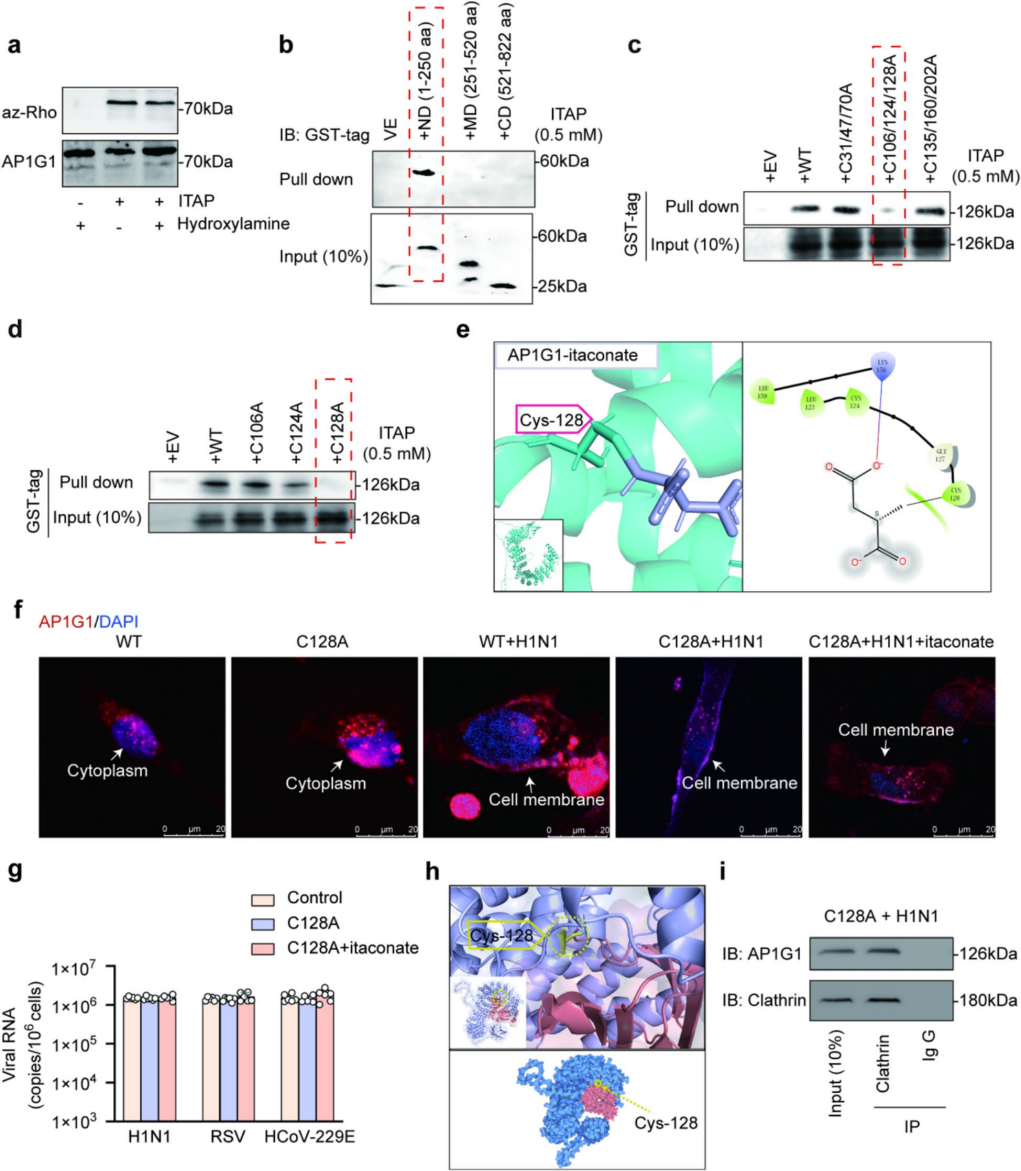
**a** Itaconate binds to AP1G1 via thioester bond. AP1G1 was incubated with 100 μM of ITAP for 1 h, followed by 5% hydroxylamine treatment. Itaconation was detected with azide-Rho click assay, *n* = 3. Itaconation on AP1G1 was found to be resistant to cleavage by hydroxylamine. **b** The binding site between AP1G1 and ITAP located within 1–250 aa. AP1G1 was synthesized in three GST-tagged fragments, including the N-domain (ND: 1–250 aa), Middle-domain (MD: 251–520 aa), and C-domain (CD: 521–822 aa). The N-domain (ND: 1–250 aa) was pulled down by ITAP. **c**, **d** The binding site between AP1G1 and ITAP located at C128. ITAP was incubated with peptides: C31/47/70A, C106/124/128A, and C135/160/202A (**c**), and peptides: C106A, C124A, and C128A (**d**). **e** Molecular docking result illustrating interaction site between itaconate and AP1G1 located at C128. **f** Immunofluorescence assay showing the subcellular localization of AP1G1 under five conditions: wild-type cells, C128A mutant cells, wild-type cells infected with H1N1 virus, C128A mutant cells infected with H1N1 virus, and C128A mutant cells infected with H1N1 virus followed by itaconate treatment. Under both uninfected and H1N1-infected conditions, C128A mutant cells exhibited behavior consistent with that of wild-type cells, indicating that the C128A mutation did not affect the subcellular localization of AP1G1. However, the C128A mutation blocked the effect of itaconate on AP1G1’ s subcellular localization. **g** Viral RNA levels in BEAS-2B cells and C128A BEAS-2B cells. Cells were infected with H1N1, RSV, and HCoV-229E viruses (MOI = 2), *n* = 6. The C128A site mutation caused itaconate to lose its ability to inhibit the invasion of three viruses. **h** Molecular docking experiments demonstrated that the Cys128 residue of AP1G1 is situated within the interaction interface between AP1G1 and clathrin. **i** Western blot assay demonstrated that the C128A site mutation did not affect the interaction between AP1G1 and clathrin

Further, it was indicated that AP1G1 is modified by Michael addition rather than by a thioester linkage, since the itaconation of recombinant AP1G1 (Fig. S3a, b) was not cleavable by hydroxylamine (Fig. 4a). To identify the binding site of itaconate on AP1G1, pull-down experiments were conducted using GST-tagged recombinant constructs of AP1G1, including the N-domain (ND: 1–250 aa), Middle-domain (MD: 251–520 aa), and C-domain (CD: 521–822 aa). The N-domain (ND: 1–250 aa) was enriched by ITAP (Fig. 4b), indicating that the itaconation occurs at cysteine residues within this region of AP1G1. However, multiple cysteine residues are present within the N-domain. To screen for the specific cysteine residue involved in the interaction, cysteines within the 1–250 aa region of AP1G1 were mutated. When Cys106, Cys124, and Cys128 were mutated to alanine in combination, the ITAP-AP1G1 interaction was attenuated, while mutation of either C106A or C124A alone had no significant effect on itaconate levels (Fig. 4c). Subsequently, Cys106, Cys124, and Cys128 were mutated individually. Mutation of Cys128 to alanine (C128A) led to a reduced interaction between ITAP and AP1G1 (Fig. 4d). Consistently, molecular docking indicated that Cys128 may serve as the covalent interaction site for itaconate (Fig. 4e), and the Lys156-Leu159 region was also implicated in the interaction, which aligns with the observed of reduced hydrolytic accessibility at these sites. These data imply that AP1G1 contains a compatible binding pocket for itaconate, mediated by Cys128 and Lys156-Leu159, with Cys128 being essential for binding.

To elucidate how itaconate binding at the Cys128 site of AP1G1 regulates AP1G1 subcellular localization and its interaction with clathrin, thereby affecting viral endocytosis, the impact of Cys128 site mutations was examined. Immunofluorescence and Western blot analyses showed that, in both BEAS-2B cells and C128A BEAS-2B cells, the distribution of AP1G1 on the cell membrane increased following viral infection, indicating that the Cys128 site mutation alone does not affect AP1G1 subcellular localization. However, in C128A BEAS-2B cells, itaconate treatment failed to retain AP1G1 in the cytoplasm, and membrane localization remained elevated, indicating that the Cys128 site mutation abolishes the regulatory effect of itaconate on AP1G1 localization (Figs. 4f, S3c). Correspondingly, no significant difference in viral load was observed between BEAS-2B cells and C128A BEAS-2B cells infected with the three viruses. Additionally, itaconate treatment did not significantly alter viral load in C128A BEAS-2B cells (Fig. 4g), indicating that the Cys128 site mutation does not intrinsically alter cellular susceptibility to the viruses but abolishes the inhibitory effect of itaconate on viral invasion.

To investigate the underlying reasons for this phenomenon, molecular docking was performed to analyze the interaction between AP1G1 and clathrin. The results indicated that Cys128 is not a direct binding residue in the AP1G1–clathrin interaction; however, Cys128 is located within the AP1G1 and clathrin interaction interface (Fig. 4h). Western blot analysis also confirmed that mutation at the Cys128 site does not disrupt the interaction between AP1G1 and clathrin (Fig. 4i).

In summary, these experiments suggest that itaconate binding at Cys128 of AP1G1 impairs the interaction between AP1G1 and clathrin, thereby reducing viral invasion. However, mutation of the Cys128 site mutation selectively prevents itaconate binding without disrupting the interaction between AP1G1 and clathrin. Therefore, the Cys128 mutation likely inhibits itaconate from binding to AP1G1. During viral infection, AP1G1 localizes to the cell membrane, interacts with clathrin, and may thereby facilitate viral invasion.

### Exploring AP1G1-interacting small molecules via thermo-proteomic analysis

**Fig. 5:**
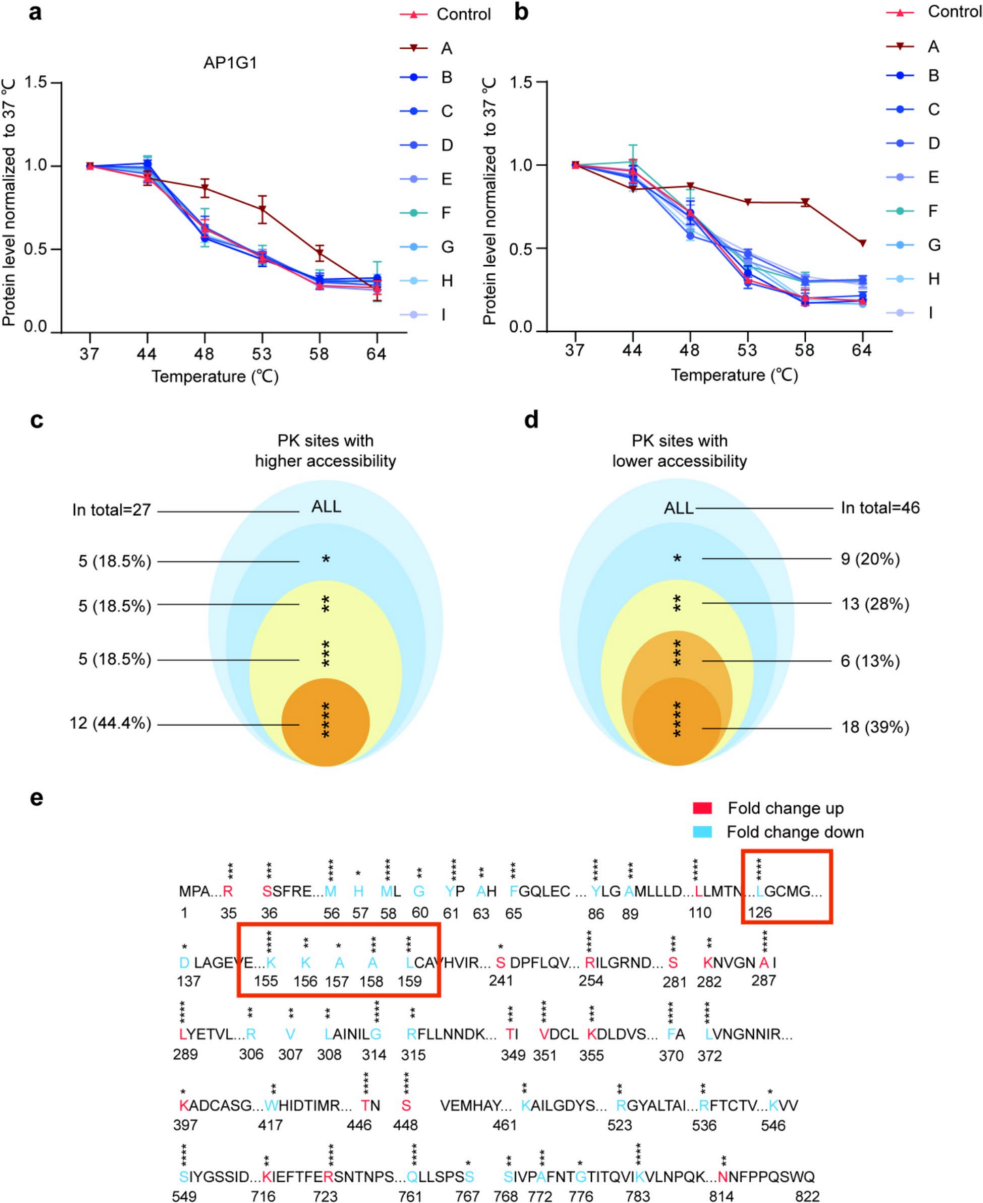
**a** The protein content change curves of AP1G1 upon incubation with traditional Chinese medicine extraction. A: Glycyrrhizae Radix et Rhizoma, B: Arctii Fructus, C: Phragmitis Rhizoma, D: Platycodonis Radix, E: Coptidis Rhizoma, F: Scutellariae Radix, G: Taraxaci Herba, H: Gardeniae Fructus, I: Forsythiae Fructus. Compared with the control group, the Glycyrrhizae Radix and Rhizoma extraction cause thermal shift in AP1G1, *n* = 3. **b** The protein content change curves of AP1G1 upon incubation with nine small molecules of the Glycyrrhizae Radix and Rhizoma extraction. A: Licochalcone B, B: Glycyrrhizic acid, C: Glycyrrhizin, D: Quercetin, E: Glycyrrhetic acid, F: Isoliquiritigenin, G: Glabridin, H: Licoricone J, I: Licocoumarin. Compared with the control group, Licochalcone B cause thermal shift in AP1G1, *n* = 3. **c**, **d** Number, classification, and status of PK sites with higher (**c**) or lower (**d**) accessibility. Asterisk symbols indicate the magnitude or condition of intensity changes: *—intensity changed by 2–3 fold; **—intensity changed by 3–10 fold; ***—intensity changed by more than 10 fold; ****—sites detectable only after (in c) or before (in d) binding or conformational change. **e** The sites in AP1G1 where the accessibility to protease K hydrolysis is upregulated or downregulated, with fold change up indicating increased accessibility, fold change down indicating decreased accessibility, *n* = 4

The inhibitory role of itaconate in viral entry has been confirmed, with its target site identified as Cys128 on AP1G1. However, the clinical application of itaconate remains limited to cellular and animal studies, as no human trials have been conducted to date. To assess the practical potential of this therapeutic approach targeting AP1G1, we sought an alternative to itaconate among antiviral traditional Chinese medicines with a long history of use. Among commonly used antiviral herbs, *Glycyrrhizae Radix et Rhizoma* was found to contain molecules that interact with AP1G1, as demonstrated by a significant shift in the thermal denaturation curve of AP1G1 (Fig. 5a). Subsequently, dozens of candidate molecules were screened during the selection process. Ultimately, Licochalcone B from *Glycyrrhizae Radix et Rhizoma* was identified as a potent AP1G1-interacting molecule (Fig. 5b).

To investigate whether Licochalcone B and itaconate share the same binding site on AP1G1, LiP-MS was employed to identify the interaction site of Licochalcone B. LiP-MS leverages changes in protease accessibility to reveal binding sites and protein conformational alterations upon molecule-protein interaction. A total of 73 semi-specific (SS) peptides were identified, of which 27 exhibited increased accessibility as PK sites, while 46 showed decreased accessibility. Peptides with increased accessibility indicate that the cleavage sites of AP1G1 become more exposed to protease digestion after treatment with Licochalcone B. Conversely, decreased accessibility suggests that these cleavage sites are either bound by compounds or concealed due to conformational changes induced by the interaction with Licochalcone B. Among the PK sites with increased accessibility, five residues exhibited a 2–3 fold intensity increase (18.5%), another five showed a stronger increase of 3–10 fold (18.5%), and five displayed an increase of more than 10 fold (18.5%). Additionally, twelve residues were detected exclusively after binding or conformational changes, accounting for 44.4% of the total (Fig. 5c). Among the sites with decreased accessibility, nine residues showed a 2–3 fold decrease (20%), thirteen exhibited a 3–10 fold decrease (28%), and six displayed a decrease greater than 10 fold (13%). Furthermore, eighteen residues were detected exclusively before binding or conformational changes, representing 39% of the total (Fig. 5d). Detailed information on the specific PK sites corresponding to each category is summarized in Table 1. These changes in PK site accessibility suggest that Licochalcone B interacts directly with AP1G1. All sites exhibiting altered protease hydrolysis accessibility were catalogued. Notably, within the N-domain (1-250aa), a shift in protease hydrolysis accessibility was observed at Leu126, which is proximal to Cys128. This observation suggests that Cys128 may serve as a potential binding site for Licochalcone B (Fig. 5e).

**Table 1:** Accessibility changes of PK sites upon binding or conformational changes. The table summarizes the number, relative intensity changes, and percentages of PK sites with altered accessibility. Specific residues are listed in the table, while overall trends are described in the text

Accessibility group	Sites (No.)	Intensity change	Percentage of total
Higher-accessibility	S241, R248, L249, R345, K397	↑ 2–3 fold	18.5%
	K282, K716, I733, Q736, N814	↑ 3–10 fold	18.5%
	R35, S36, S281, T349, K355	↑ > 10 fold	18.5%
	K108, L110, M129, K186, R254, A287, L289, M297, V351, T446, S448, R723	Detected only after binding	44.4%
Lower-accessibility	H57, D137, A157, H163, K546, S553, S554, S767, G776	↓ 2–3 fold	20%
	G60, A63, K156, R306, V307, L308, L313, R315, W417, K461, R523, R536, S768	↓ 3–10 fold	28%
	F65, A89, A158, A159, L322, A772	↓ > 10 fold	13%
	M56, M58, Y61, E69, K72, Y86, L126, R136, K155, R166, G314, F370, L372, F531, S549, R562, Q761, K783	Detected only before binding	39%

### Licochalcone B is an itaconate substitute for reducing host susceptibility to respiratory virus

**Fig. 6:**
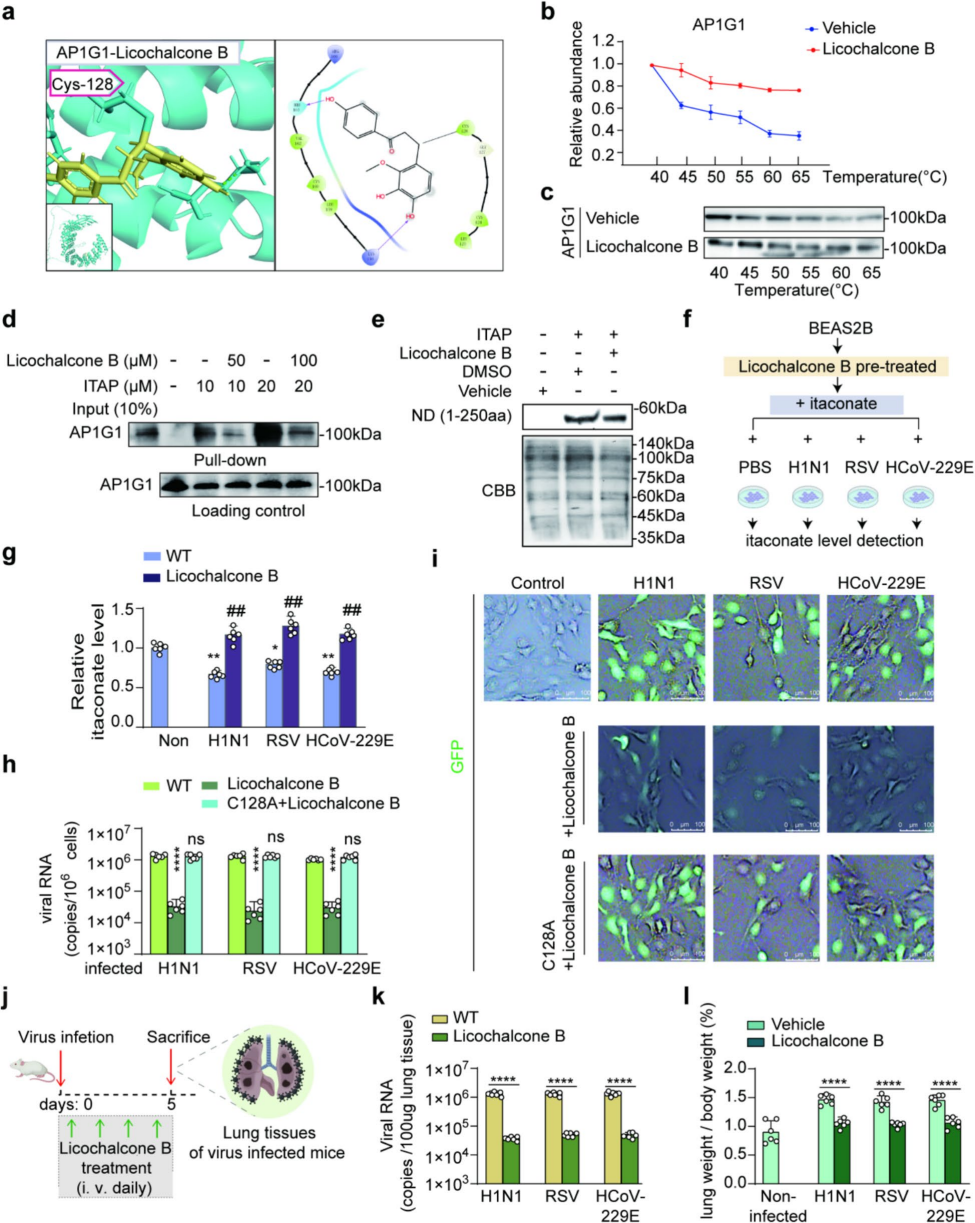
**a** Molecular docking result illustrating interaction site between Licochalcone B and AP1G1 located at C128. **b**, **c** Licochalcone B (100 μg/ml) treatment significantly altered the thermal stability of AP1G1. Thermal shift curve of AP1G1 was determined by CETSA-Western blot at 40, 45, 50, 55, 60, and 65 ℃. Western blot image (**c**) and quantitative analysis (**b**) showing increased thermal stability of AP1G1 in the Licochalcone B-treated group compared to the vehicle, *n* = 3. **d** Licochalcone B competitively binding on AP1G1 and impede the pull-down efficacy of ITAP. BEAS-2B cell lysates were incubated with ITAP (10 μM or 20 μM) upon the competition of itaconate (50 μM or 100 μM) under native conditions. After incubation, ITAP-bound proteins were captured with biotin-click and streptavidin-conjugated beads, *n* = 3. **e** Competitive binding of Licochalcone B to N-domain (1–250 aa). BEAS-2B cell lysates were incubated with 1 mM ITAP and then with 100 μg/ml of Licochalcone B for 24h, *n* = 3. **f**, **g** Licochalcone B treatment preserves the itaconate content in BEAS-2B cells. Cells were first pretreated with licochalcone B (200 μM, 24 h) and then exposed to H1N1, RSV, and HCoV-229E virus infections and cultured in itaconate-supplied medium for 24 h (**f**). Itaconate levels (**g**) were detected in BEAS-2B cells (MOI = 2), *n* = 6, **p < 0.01, ^##^*p* < 0.01. **h**, **i** C128 of AP1G1 serves as a key target of Licochalcone B in inhibiting virus invasion. Viral RNA levels in BEAS-2B cells that were infected with H1N1, RSV, or HCoV-229E viruses (MOI = 2, 24 hpi) under three conditions: vector control, vector + Licochalcone B, C128A + itaconate. Viral RNA copies were quantified by qPCR, *n* = 6, ****p < 0.0001 (**h**). BEAS-2B cells were infected with GFP-tagged viruses of H1N1, RSV, and HCoV-229E (MOI = 2, 24 hpi). The GFP signal was attenuated in the sh-AP1G1 group compared with the WT group, indicating reduced virus invasion, *n* = 3. Scale bar = 100 μm (**i**). **j** Schematic of the experimental design. Mice were infected intranasally with three types of viruses—H1N1, RSV, and HCoV-229E—at a dose of 100 TCID_50_ in a total volume of 40 µL, and then treated with Licochalcone B via tail vein injection (3 mg/kg, daily) for 5 days. Lung tissues were collected for analysis. **k** Effect of Licochalcone B on viral RNA levels in the lung tissues of virus-infected mice, *n* = 6, ****p < 0.0001. **l** Effect of Licochalcone B on lung weight/body weight ratio in virus-infected mice, *n* = 6, ****p < 0.0001

To investigate whether Licochalcone B can functionally substitute for itaconate, we examined the binding interaction between Licochalcone B and the Cys128 site of AP1G1, as well as its antiviral activity. Molecular docking results suggested a specific interaction between Licochalcone B and protonated His163 and Lys156 within a broad interface spanning K156 to R166 (Fig. 6a). The binding of Licochalcone B to AP1G1 was confirmed by altered thermal stability profiles of AP1G1 (Fig. 6b, c). Importantly, Licochalcone B competitively inhibited the binding of ITAP to AP1G1 in situ in BEAS-2B cells (Fig. 6d). Additionally, Licochalcone B competed with itaconation on the N-domain (1-250aa) of AP1G1 (Fig. 6e). In BEAS-2B cells pretreated with Licochalcone B and subsequently infected with respiratory tract viruses (Fig. 6f), Licochalcone B significantly restored intracellular itaconate levels (Fig. 6g). These findings suggest that Licochalcone B may serve as an effective substitute for itaconate during respiratory tract virus infection. Furthermore, Licochalcone B was detected in both the blood and lung tissues of mice (Fig. S4), supporting its potential as a therapeutic candidate for respiratory tract infections. Administration of Licochalcone B reduced viral genome copies in vitro, resulting in overall reduction exceeding 95% compared with untreated controls (Fig. 6h, i). In summary, Licochalcone B appears to exert effects similar to those of itaconate by targeting Cys128 of AP1G1 to reduce host susceptibility to respiratory viruses.

### Licochalcone B reduces lung viral loads and alleviates virus-induced lung injury in vivo

To evaluate the effects of Licochalcone B on H1N1, RSV, and HCoV-229E infections in mouse lungs (Fig. 6j), viral RNA levels and the lung weight-to-body weight ratio (lung index) were measured. Analysis of viral RNA levels in the lungs revealed that Licochalcone B significantly reduced the number of viral particles invading lung tissues compared to the control group, indicating decreased viral load (Fig. 6k). Moreover, the lung index, an indicator of lung tissue inflammation and damage [36], was significantly lower in the Licochalcone B-treated group than in the control group, suggesting that Licochalcone B alleviates virus-induced inflammation and lung injury (Fig. 6l). Taken together, Licochalcone B appears to have a promising effect in mitigating virus-induced lung injury by reducing host susceptibility to respiratory viruses.

## Discussion

**Fig. 7:**
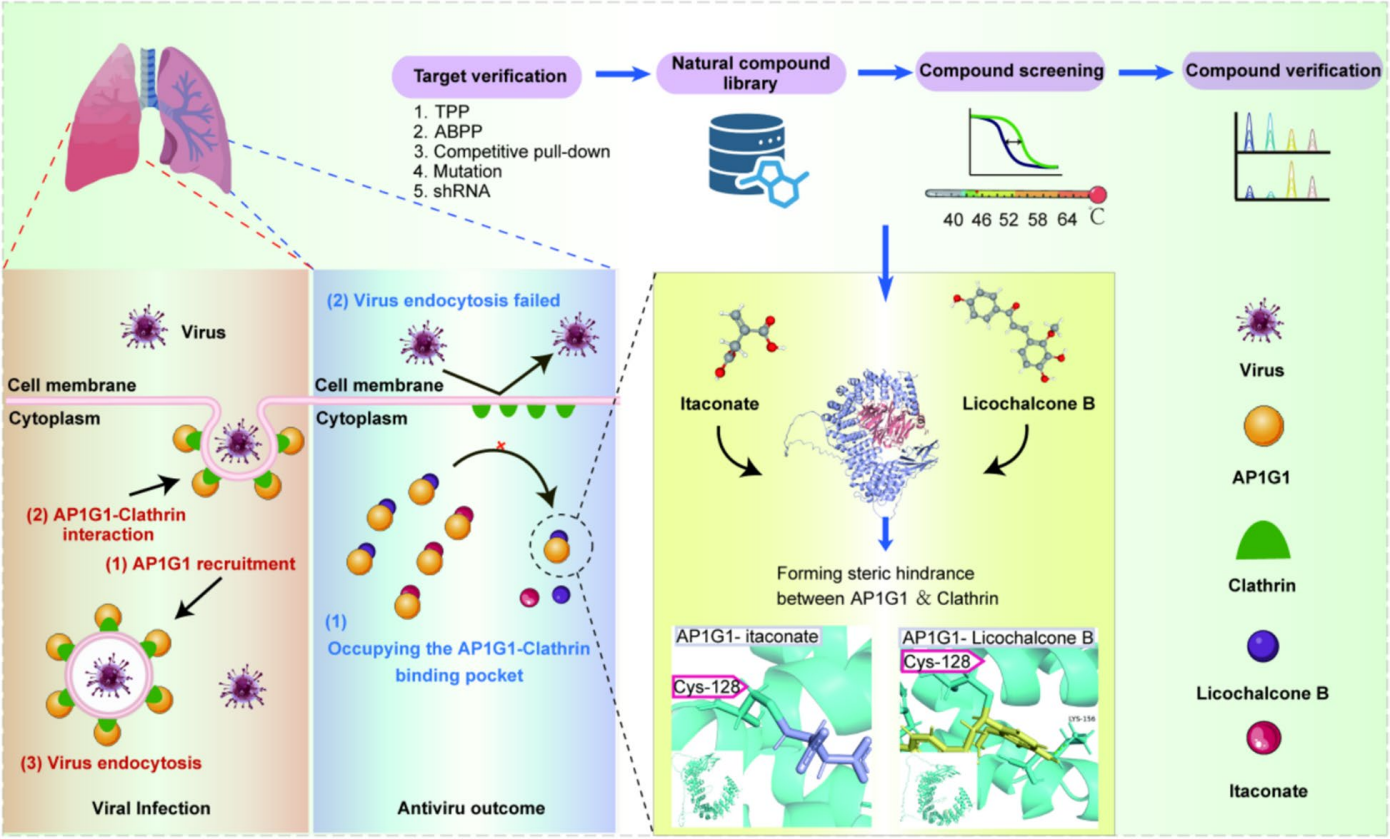
Upon the invasion of respiratory viruses, AP1G1 undergoes membrane translocation and interacts with clathrin to facilitate the endocytosis of the virus by host cells. Itaconate and drug-screened Licochalcone B can disrupt the membrane translocation of AP1G1 by binding to Cys128, thereby reducing the interaction between AP1G1 and clathrin and diminishing the invasion of respiratory viruses

Endogenous metabolites such as itaconate participate in diverse host-virus interactions and influence the progression of infectious diseases [7, 8, 37–39]. As an anti-inflammatory and antibacterial metabolite, itaconate exerts its actions by covalently modifying proteins that are integral to the inflammatory response and host defense [28]. Itaconate analogues, including DI and 4-OI, have been reported to attenuate pulmonary inflammation during respiratory viral infections [12, 40]. The broad-spectrum anti-inflammatory, antiviral, and immunomodulatory effects of itaconate and its analogs have been linked to the regulation of endocytic processes [41–45]. Endocytosis is not only a pathway for viruses to enter host cell, but is also essential for viral replication [25]. Clathrin adaptor proteins are key factors in mediating endocytosis, facilitating the invasion of various viruses, including SARS-CoV-2, Ebola, HIV, and Hepatitis B [46–49]. Through bidirectional genome-wide CRISPR screening, AP1G1 was identified as a crucial host-dependent factor for the viral entry of SARS-CoV-2, where it facilitates viral particles internalization and contributes to the pathogenesis of COVID-19, MERS-CoV, and seasonal HCoVs [27]. These findings suggests that AP1G1 represents a promising target for host-directed antiviral therapy. However, strategies to prevent virus entry by modulating AP1G1 remains largely unexplored. The present study identifies Cys128 of host proteins AP1G1 as a critical target for inhibiting respiratory viral invasion. AP1G1 plays a pivotal role in virus endocytosis [27, 50, 51], facilitating the subcellular localization of viral proteins to the so-called "viral factory" [52]. Itaconate covalently modifies the Cys128 residue of AP1G1, thereby disrupting its native interactions and impairing its function. Specifically, the covalent occupation of Cys128 by itaconate interferes with AP1G1 membrane translocation, consequently weakening its interaction with clathrin, a key mediator of host cell endocytosis, and thereby limiting the invasion of respiratory viruses (Fig. 7). These findings underscore the strategic importance of Cys128 within AP1G1 as a critical target for blocking viral invasion, highlighting the precise molecular mechanism through which itaconate modulates host-virus interactions at the molecular level. The study elucidates a previously unrecognized antiviral mechanism of itaconate that operates independently of its classical immunomodulatory function. This research broadens the current understanding of the regulatory network linking metabolite-protein modifications to viral infections processes. This study provides the first evidence that post-translational modification of AP1G1, specifically Cys128 itaconation, directly influence viral invasion. This finding establishes a new paradigm for investigating host-virus interactions.

Currently, there are numerous reports on the antiviral effects of various natural drug molecules. For instance, plant-derived compounds such as trans-δ-viniferin derivatives, pheophorbide A (Pba), and Echinaforce® exhibit broad-spectrum antiviral activity and effectively inhibit enveloped viruses, including influenza virus, SARS-CoV-2, and herpes simplex virus 2. With the discovery of the broad-spectrum antiviral target Cys128, screening and identifying promising lead compounds from natural medicinal molecule libraries has become particularly important [53–55]. To enhance the translational potential of the Cys128 targeting strategy against pathogen invasion, this study combined thermal proteomic profiling with LiP-MS spectrometry. Through this approach, an itaconate analogue—Licochalcone B—was identified from the well-known antiviral traditional Chinese medicine *Glycyrrhizae Radix et Rhizoma* (licorice). Preliminary pharmacokinetic analyses in mice revealed that Licochalcone B possesses moderate oral bioavailability and a favorable safety profile at effective doses [56–58]. No acute toxicity or significant organ damage was observed at concentrations achieving antiviral efficacy, laying the foundation for further translational research. Notably, while this study focused on three representative respiratory viruses (H1N1, RSV, and HCoV-229E), the conserved nature of AP1G1 suggests that its Cys128 site may serve as a viable antiviral target across a broader range of viruses, including SARS-CoV, MERS-CoV, and enteroviruses. Future studies should systematically assess the inhibitory effects of Licochalcone B in these and other viral systems. Further investigation confirmed that Licochalcone B specifically targets the Cys128 site within AP1G1, effectively inhibiting viral replication and propagation. Compared to oseltamivir, Licochalcone B targets a conserved host pathway, offering potentially greater durability and reduced resistance risk, along with added anti-inflammatory benefits. This discovery not only has a druggable host target been discovered, but also provides a successful example of screening host-directed antiviral molecules from natural compounds, with significant implications for both basic research and clinical translation.

Despite the promising findings, several limitations must be acknowledged. First, Licochalcone B remains in the preclinical research phase, and its pharmacokinetic properties, including metabolism and bioavailability, warrant further investigation. Itaconate is characterized by rapid metabolism and low bioavailability, which may similarly affect the efficacy of Licochalcone B. Therefore, optimizing the formulation and delivery strategies of Licochalcone B will be essential for advancing it as a clinically viable lead compound. Furthermore, a limitation of the current study is that the applicability of this mechanism was not investigated at the animal level. Hence, future studies are warranted to validate these findings, initially in appropriate animal models and subsequently in clinical settings. Such research will be essential for advancing the understanding of host defense mechanisms against viral infections.

In summary, the present study identifies Cys128 of the host protein AP1G1 as a crucial target for inhibiting respiratory viral invasion. Itaconate and its analogue, Licochalcone B, were shown to specifically modify this residue, disrupting AP1G1 membrane translocation and clathrin interactions, and consequently suppress viral endocytosis and replication. This work elucidates a previously unrecognized antiviral mechanism independent of classical immunomodulatory pathways and establishes proof of concept for the identification of host-directed antiviral agents derived from natural products. Given the conserved nature of AP1G1, targeting Cys128 may confer broad-spectrum antiviral potential and lay a solid foundation for future translational studies aimed at developing safe and effective antiviral therapies.

## Materials and methods

### Materials and reagents

Chinese herbal medicines and standard compounds were purchased from China National Medicinal Materials Corporation and Chengdu Herbpurify. Chemicals and antibodies were obtained from Fisher Scientific, Sigma-Aldrich, Novus, and other commercial suppliers.

### Animal experiments

Male KM mice were infected intranasally with H1N1, RSV, or HCoV-229E viruses. Licochalcone B (3 mg/kg) or saline was administered daily via tail vein injection for 5 days. Lung and blood samples were collected on day 5 post-infection.

### Cell culture and virus infection

BEAS-2B cells (Immocell Biotechnology Co.,Ltd.) were maintained in DMEM with 10% FBS. Viral infections were performed under BSL-2 conditions using H1N1, RSV, or HCoV-229E strains.

### Itaconate identification and quantification

Itaconate was extracted from lung tissue and analyzed using UHPLC-Q Exactive Orbitrap MS in HILIC mode. Quality control samples were included during the sequence.

### Western blotting and RT-qPCR

Proteins were detected by Western blot using specific antibodies. Viral RNA was quantified by RT-qPCR, and relative expression was calculated by the 2^–ΔΔCT method.

### Cloning, recombinant protein preparation and GST pull-down

AP1G1 domains and mutants were cloned into His- or GST-tagged vectors. Recombinant proteins were expressed in E. coli and purified. Protein interactions were assessed by GST pull-down.

### Itaconate-probe synthesis, labeling and protein enrichment

An itaconate-derived probe was synthesized and used to treat cells. Labeled proteins were conjugated to biotin-azide via click chemistry and enriched with streptavidin beads.

### Hydroxylamine cleavage assay

Recombinant AP1G1 was incubated with itaconate-alkyne, treated with hydroxylamine, and labeled with rhodamine-azide. Modification was detected by SDS-PAGE.

### Biotin click assay

Biotin-streptavidin pull-down was performed after itaconate-probe labeling. Competition assay were conducted with itaconate or Licochalcone B pretreatment.

### Molecular docking

Covalent docking using Schrödinger software was performed to investigate the binding of itaconate or Licochalcone B to AP1G1 (PDB ID: 1IU1), as well as the interaction between AP1G1 and clathrin (PDB ID: 6E4L).

### Subcellular localization

Cellular fractionation and confocal microscopy were used to analyze AP1G1 localization.

### Limited proteolysis-mass spectrometry (LiP-MS)

AP1G1 was incubated with licorice extract or control, subjected to limited proteolysis, and analyzed by LC–MS/MS to map structural changes.

### Thermal proteome profiling (TPP)

TPP-ELISA and TPP-WB were used to evaluate thermal stability shifts of AP1G1 upon ligand binding.

### Statistical analysis

Data are shown as mean ± SD. Significance was determined by t-test (P* < 0.05, P** < 0.01, P*** < 0.001, P**** < 0.0001).

## Supplementary Information

Supplementary Material 1

## Data Availability

The data and materials that support the findings of this study are available from the corresponding author upon reasonable request. Some data and materials may not be made available because of privacy or ethical restrictions.
