# Supplementary material for: Itaconate reduces viral endocytosis by targeting Cys128 of the adaptor-related protein complex 1 gamma 1 subunit in the host, providing a novel target for antiviral drug development
Source: Mol Biomed. 2025 Nov 10;6:105. doi: 10.1186/s43556-025-00348-6 (PMC12602806; doi:10.1186/s43556-025-00348-6)
Supplement: Supplementary file 1 — Supplementary Material 1 [file 43556_2025_348_MOESM1_ESM.docx]

**Itaconate reduces viral endocytosis by targeting Cys128 of the adaptor-related protein complex 1 gamma 1 subunit in the host, providing a novel target for antiviral drug development**

Xinqi Deng^1^†*, Heng Chen^2^†, Zhixing Huang^2^†, Rongge He^2^, Qinling Rao^2^, Luni Xu^2^, Zijian Xu^2^, Naixuan Zhao^2^, Yeqing Peng^2^, Muxuan Li^2^, Xi Liu^2^, Tao Ma^2^*, Xiaolan Cui^1^*, Chunguo Wang^2^*

1. Institute of Chinese Materia Medica, China Academy of Chinese Medical Sciences, Beijing 100700, China.

2. Beijing University of Chinese Medicine, Beijing 102488, China.

* Corresponding authors:

xqdeng@icmm.ac.cn (Xinqi Deng)

201701033@bucm.edu.cn(Tao Ma);

cuixiaolan2812@126.com(Xiaolan Cui); chunguowang@bucm.edu.cn(Chunguo Wang);

† These authors contribute equally to this work.

**Keywords:** itaconate, post-translational modification, virus, AP1G1, Clathrin

**SUPPLEMENTARY INFORMATION**

1. **MATERIALS AND METHODS**

**9.1. Materials and Reagents**

Chinese herbal medicines (Arctii Fructus, Phragmitis Rhizoma, etc.) and standard substances (Licochalcone B, Glycyrrhizic acid, etc.) were purchased from China National Medicinal Materials Corporation and Chengdu Herbpurify Co., Ltd. Chemicals such as methanol, formic acid, and acetonitrile were from Fisher Scientific. Trypsin (modified, sequencing grade), Urea (Promega). Streptavidin magnetic beads (MCE). Native Lysis Buffer, BCA Protein Assay Kit (Beijing Solarbio Science & Technology Co.,Ltd.). Primary antibodies of AP1G1 (novus, NBP3-15727). The other chemicals were purchased from Sigma–Aldrich unless stated otherwise.

**9.2. Animal Experiments**

Male specific pathogen-free Kunming (KM) mice (20-30g) from Vital River (Beijing) Laboratory Animal Technology Co., LTD were housed under standard conditions. Mice were randomly divided into groups and infected intranasally with 100 TCID50 of H1N1, RSV, or 229E viruses (40 µL total volume), except for the control group. Based on preliminary dose optimization, Licochalcone B (3 mg/kg) or an equal volume of saline (vehicle control) was administered via tail vein injection daily for 5 days, starting from the day of infection. Blood and lung tissues were collected on day 5 for analysis. All procedures were approved by the China Academy of Chinese Medical Sciences (Ethics Approval No. 2022D052).

**9.3. Cell Cultures and Virus Infection**

BEAS-2B cells (Immocell Culture Collection) were maintained in DMEM supplemented with 10% FBS and 1% Penicillin-Streptomycin. Influenza A (H1N1)/2009 TC isolate, human respiratory syncytial virus (RSV, strain 18537), and human coronavirus 229E (strain 229E) were handled under biosafety level 2 conditions.

**9.4. Itaconate Identification and Quantification**

Small molecules were extracted with methanol/acetonitrile solution (3:1, v/v) from lung tissue and detected in HILIC mode with UPLC BEH Amide column (2.1 mm×100 mm, 1.7 μm). Analysis was performed on a Thermo Scientific Vanquish UHPLC coupled to a Q Exactive Orbitrap MS system equipped with an HESI-II ionization source. To validate the analytical methodology, pooled quality control (QC) samples were prepared by mixing sample from each group. Five QC samples were analyzed before sample sequencing, and during the analysis of the sample sequence, one QC sample was run after every five injections[1,2]. The operating parameters were as follows: spray voltage, 3.5 KV; sheath gas pressure, 35 arb; auxiliary gas pressure, 10 arb; capillary temp, 300 °C; ion source temp, 350 °C; scan modes, MS (Full Scan, m/z100-1200) and data-dependent acquisition MS2 (resolution 17,500, normalized collision energy 35 eV, stepped normalized collision energy 30 and 40 eV) and scan range, m/z 80–1200. The mobile phases were combined with 0.1% formic acid in water (A) and 0.1% formic acid in acetonitrile (B).

UHPLC HILIC gradient conditions: 0-10 min, 8-30% (A)；10-12 min, 30 -30 % (A) ；12-13 min, 30-8% (A)；13-15 min, 8-8% (A).

**9.5. Western Blotting and RT-qPCR Assay**

For Western blotting, protein concentrations were determined using a BCA assay kit. Equal amounts of protein (1 μg/μL) were separated by 12% SDS-PAGE and transferred to PVDF membranes. After blocking with 5% nonfat milk, membranes were incubated with primary antibodies followed by HRP-conjugated secondary antibodies. Protein bands were visualized using a chemiluminescence imager.

For viral RNA quantification, total RNA was extracted with TRIzol and reverse transcribed into cDNA using random primers and M-MLV reverse transcriptase. Virus-specific RNA levels (H1N1, RSV, 229E) were measured by qPCR using commercial kits (Shanghai ZJ Bio-tech) on an Applied Biosystems 7500 system. The relative fold change was calculated with the formula for relative FC=2^‐ΔΔCT^.

**9.6. Cloning, Recombinant Protein Preparation and GST Pull-Down Assay**

For gene knockdown, lentiviral particles expressing AP1G1-specific shRNAs (shRNA-1: 5’-GGAATAATATCCGAGGCATGA-3’; shRNA-2: 5’-GCATTGTCCCAGCATTTAACA-3’) or scramble control were produced in 293T cells. BEAS-2B cells were infected and selected with puromycin (1 µg/mL). For overexpression, GST-tagged AP1G1 wild-type and C128A mutant were cloned into the pGWLV11-new vector and packaged as lentiviruses.

For recombinant protein expression, the cDNA sequences encoding the N-terminal (ND, aa 1-250), middle (MD, aa 251-520), and C-terminal (CD, aa 521-822) domains of AP1G1 (Uniprot-derived) were inserted into a His-tagged pET28a plasmid. The recombinant plasmids were transformed into Escherichia coli BL21(DE3) cells. Protein expression was induced with IPTG at 16 °C when OD600 reached 0.5-0.6. Following induction, cells were harvested, sonicated, and the His-tagged proteins were purified using nickel-nitrilotriacetic acid (Ni-NTA) beads. In addition, full-length AP1G1 sequences were cloned into an N-Sumo-affinity tag containing vector (pSumo-mut, Novagen). E. coli BL21(DE3) cells were cultured in LB medium supplemented with 200 µg/mL kanamycin at 37 °C until OD600 reached 0.6-0.8. Overexpression was induced with 0.2 mM IPTG at 37 °C overnight. Cells were harvested by centrifugation, lysed by ultrasonication, and clarified by centrifugation. Soluble fractions were purified using Ni-IDA resin (Novagen), followed by overnight dialysis for further purification.

To investigate the role of specific cysteine residues, a series of GST-tagged AP1G1 fragments (amino acids 1-250) containing point mutations were constructed, including wild-type (WT) AP1G1, the triple mutants C31/47/70A, C106/124/128A, C135/160/202A, and the single mutants C106A, C124A, and C128A. All constructs were cloned into the pCDH1-MCS-coGFP vector for expression. For GST pull-down assays, cell lysates expressing GST-tagged proteins were incubated with GST antibodies and A/G agarose beads according to the manufacturer’s instructions. After extensive washing, bound proteins were eluted and analyzed by SDS-PAGE and immunoblotting.

**9.7. Itaconate-Probe Synthesis, Labeling, and Protein Enrichment**

Probe Synthesis: Itaconate anhydride and 7-Octyn-1-ol (1:1, w/w) were heated at 110 °C for 4 hours. After cooling, the mixture was precipitated in hexane. The resulting solid was purified by silica column chromatography using a gradient of 5-6% methanol in methylene chloride to yield the final itaconate-probe.

Cellular Labeling and Protein Pull-Down: BEAS-2B cells were treated with the itaconate-probe (100 µM in DMEM) for 24 hours. Cells were then lysed, and the lysates were subjected to CuAAC click chemistry with biotin-azide to label itaconate-bound proteins. The reaction mixture included 0.1 mM biotin-azide, 1 mM TCEP, 0.2 mM TBTA, and 1 mM CuSO4, and was incubated for 3 hours at room temperature. Proteins were precipitated with acetone at -80°C, and the biotinylated proteins were enriched using streptavidin beads. After extensive washing to remove non-specific binders, the captured proteins were digested with trypsin using the FASP method for subsequent analysis.

**9.8. Hydroxylamine Cleavage Assay**

The recombinant proteins AP1G1 was suspended in lysis buffer respectively. The protein concentration was determined by using the BCA protein assay kit. 50 µL of recombinant protein (1 mg/ml) was incubated with 100 μM itaconate-alkyne at 37 °C for 1 h.

For hydroxylamine cleavage assay, hydroxylamine was added to 5% concentration. The protein solution was then precipitated by 200 µL methanol, 50 µL chloroform and 150 µL Milli-Q water. The precipitated proteins were centrifuged at 8000 g for 5 min at 4 °C and washed twice with 500 µL cold methanol. Then precipitated proteins were resuspended in 50 µL PBS containing 0.4% SDS, 1 mM CuSO_4_, 100 μM TBTA ligand, 100 μM Rhodamine-azide, and 1 mM TCEP for 1 h at room temperature. The protein solution was then precipitated again to remove redundant reagent. The samples were resolved on 10% SDS-PAGE gels for further imaging.

**9.9. Biotin Click Assay of Itaconate-Probe**

The recombinant proteins AP1G1 solution were treated with itaconate-probe for 4 h. The protein solution was then precipitated by 200 µL methanol, 50 µL chloroform and 150 µL Milli-Q water. The precipitated proteins were centrifuged at 8000 g for 5 min at 4 °C and washed twice with 500 µL cold methanol. Then precipitated proteins were resuspended in 50 µL PBS containing 1.2% SDS. 100 µL streptavidin beads were washed for three times with 1 mL PBS, and added to the protein solution. The resulting solution was incubated for 4 h at RT, followed by washing with 1 mL PBS for three times, and 1 mL distilled water for three times. The resulting beads were resuspended in 500 µl loading buffer and boiled for 5 min for western-blotting.

For competitive binding pull down assay, BEAS-2B cells were pre-treated with itaconate or Licochalcone B for 1 h, followed by adding the itaconate-probe or DMSO in fresh medium. After incubation for 4 h, the soluble proteins were extracted to perform click reaction. The pull down experiment was carried out through incubation with streptavidin coupled beads at RT for 4 h with gentle rotation. The enriched proteins were eluted from beads with 1×SDS-loading bufer by boiling at 100 °C for 10 min. The collected supernatants were subjected to western blotting.

**9.10. Molecular Docking Analysis**

To study the effect of covalent docking [3], itaconate or Licochalcone B was docked to the model protein AP1G1 (PDB ID: 1IU1) using the Covalent Dock program within the Schrödinger software suite. Additionally, docking analyses were conducted between AP1G1 and clathrin (PDB ID: 6E4L) to investigate their potential protein-protein interactions. The top five docked poses were retained in the docking output, but only the pose with the best binding affinity was reported. All other parameters were set to their default values.

The schematic diagram illustrating the interaction between AP1G1 and clathrin, rendered in the Goodsell style, was created using Illustrate software developed by [David S Goodsell](https://pubmed.ncbi.nlm.nih.gov/?term=Goodsell+DS&cauthor_id=31519398) et al [4].

**9.11. Subcellular Localization Analysis**

Cellular fluorescence imaging was performed following the manufacturer's protocol. Images were captured using a Leica SP8 confocal laser scanning microscope and processed with LAS X software.

Cells were washed and lifted with PBS containing 2.5 mM EDTA, followed by washing in hypotonic buffer. After centrifugation, cells were resuspended in hypotonic buffer and incubated on ice for 30 min. Cell membranes were disrupted by passing through a 26-gauge needle. Nuclei and debris were removed by centrifugation at 2,500 g for 5 min. The post-nuclear supernatant was further centrifuged at 17,000 g for 30 min to separate membrane (pellet) and cytosolic (supernatant) fractions. The membrane pellet was washed with hypotonic buffer. Both fractions were prepared with SDS loading buffer for western blot analysis, using tubulin as a cytosolic marker.

**9.12. Limited Proteolysis-Mass Spectrometry (LiP-MS) Assay**

AP1G1 protein was incubated with licorice extract (drug) or water (control) at room temperature for 2 hours.Following limited proteolysis, samples were reduced with 15 mM DTT (56 °C, 30 min) and alkylated with 40 mM iodoacetamide (25 °C, 30 min), followed by a second treatment with 25 mM DTT (37 °C, 25 min). After vacuum drying, samples were treated with 0.1 μg/μL CNBr in 90% formic acid at 37°C for 16 hours in the dark. After incubation and drying, the peptide samples were subjected to tryptic digestion (1:50 trypsin-to-protein ratio) in a denaturing buffer (5% sodium deoxycholate, 2 M urea, 50 mM ammonium bicarbonate, pH 8.0) at 37°C for 16 hours. The reaction was quenched with TFA, and peptides were desalted using C18 columns, eluted with 90% acetonitrile/0.2% TFA, and freeze-dried.

Peptides were separated on a C18 column using an EASY-nLC 1200 system. A 120-minute gradient was used: mobile phase B (acetonitrile/0.1% formic acid) was increased from 2% to 8% in 2 min, then to 35% over 110 min, rapidly to 95% in 3 min, and held at 95% for 5 min. MS data were acquired on a Q Extractive HF mass spectrometer in data-dependent acquisition (DDA) mode. Full MS scans were performed at 60,000 resolution (m/z 350-1500), and the top 15 precursors were selected for HCD-MS/MS.

Spectral Library Generation: DDA data from all replicates were aggregated and searched against a human protein database using Spectronaut. Cysteine carbamidomethylation was set as a fixed modification. Methionine oxidation, protein N-terminal acetylation, and the C-terminal homoserine lactone conversion from CNBr cleavage were set as variable modifications. A project-specific spectral library was generated with a 1% FDR threshold.

DIA Data Analysis: DIA data from drug-treated and control samples were searched against the project-specific spectral library. Search parameters included SS digestion by trypsin and CNBr, and the same modification settings as for library generation. Cross-run normalization was enabled.

Protein Kinase (PK) Site Accessibility Assignment: SS peptides represent PK cleavage sites. A significant change in site accessibility is defined as an average SS peptide fold change of >2 or <0.5 (p < 0.05) in drug vs. control comparisons. The following rules assign a final ratio to each PK site: (1) Single Peptide: The peptide's fold change represents the site. (2) Multiple Concordant Peptides: The ratio from the peptide with the smallest p-value is used. (3) Multiple Discordant Peptides: The site is considered invalid and removed. (4) Consistent Detection under One Condition: The site is assigned a significant change (ratio = 2). (5) Mixed Significant/Consistent Peptides: The ratio from the significant peptide with the smallest p-value is used.

**9.13. TPP Assay**

TPP-ELISA assay: The extract of traditional Chinese medicine (centrifuged and filtered through a microporous membrane) or small molecular compounds from licorice were incubated with recombinant AP1G1 protein (1 mg/ml) for 30 minutes. Each group is divided into six aliquots, which are heated at gradient temperatures of 37, 44, 48, 53, 58, and 64°C for 3 minutes, followed by an additional incubation at room temperature for 3 minutes. After centrifugation at 22,000 g for 2 hours, an equal volume of supernatant was collected for the detection of AP1G1 protein content. ELISA was performed using a commercial kit (Genscript Biotech, Product No. L00411) according to the manufacturer's instructions. Briefly, GST standards and the six test samples were added to the wells of the capture plate. After the addition of 1× biotin-labeled GST antibody, the plate was incubated at 37 °C for 30 minutes. The plate was then washed, followed by the addition of HRP-labeled streptavidin and a subsequent incubation at 37 °C for 15 minutes. After another wash, a chromogenic substrate was added, and the plate was allowed to develop in the dark at room temperature for 15-20 minutes. Finally, the stop solution was added, and the absorbance was immediately measured at 450 nm.

TPP-WB assay: BEAS-2B cells were grown to 80% confluence, washed with PBS for three times and lysed by sonication in the ice and cell lysates were collected by centrifugation (20,000 g, 30 min) at 4 °C to remove the debris. 100 μg/ml of Licochalcone B, itaconate (200 μM) or DMSO were added to the lysates, samples were mixed and incubated for 15 min. Treated and non-treated lysates were divided into six aliquots, each 50 µL in PCR tubes. The aliquots were individually heated at different temperatures. After the heat treatment the cell lysates were completely transferred to polycarbonate tubes and centrifuged at 100,000 g, 4 °C for 25 min. For immunoblotting analysis, 16 µL of each supernatant was added with 4 µL of 5x loading buffer and incubated for 5 min at 95 °C. For MS protein quantification, proteins were processed and analyzed as described above.

**9.14. Statistical Analysis**

All data are presented as mean ± standard deviation (SD), with n = 6 for each group.Statistical significance was determined using t-tests. Significance levels are indicated as follows: ns (not significant), P < 0.05 (*), P < 0.01 (**), P < 0.001 (***), and P < 0.0001 (****).

1. **SUPPLEMENTARY FIGURES**

**
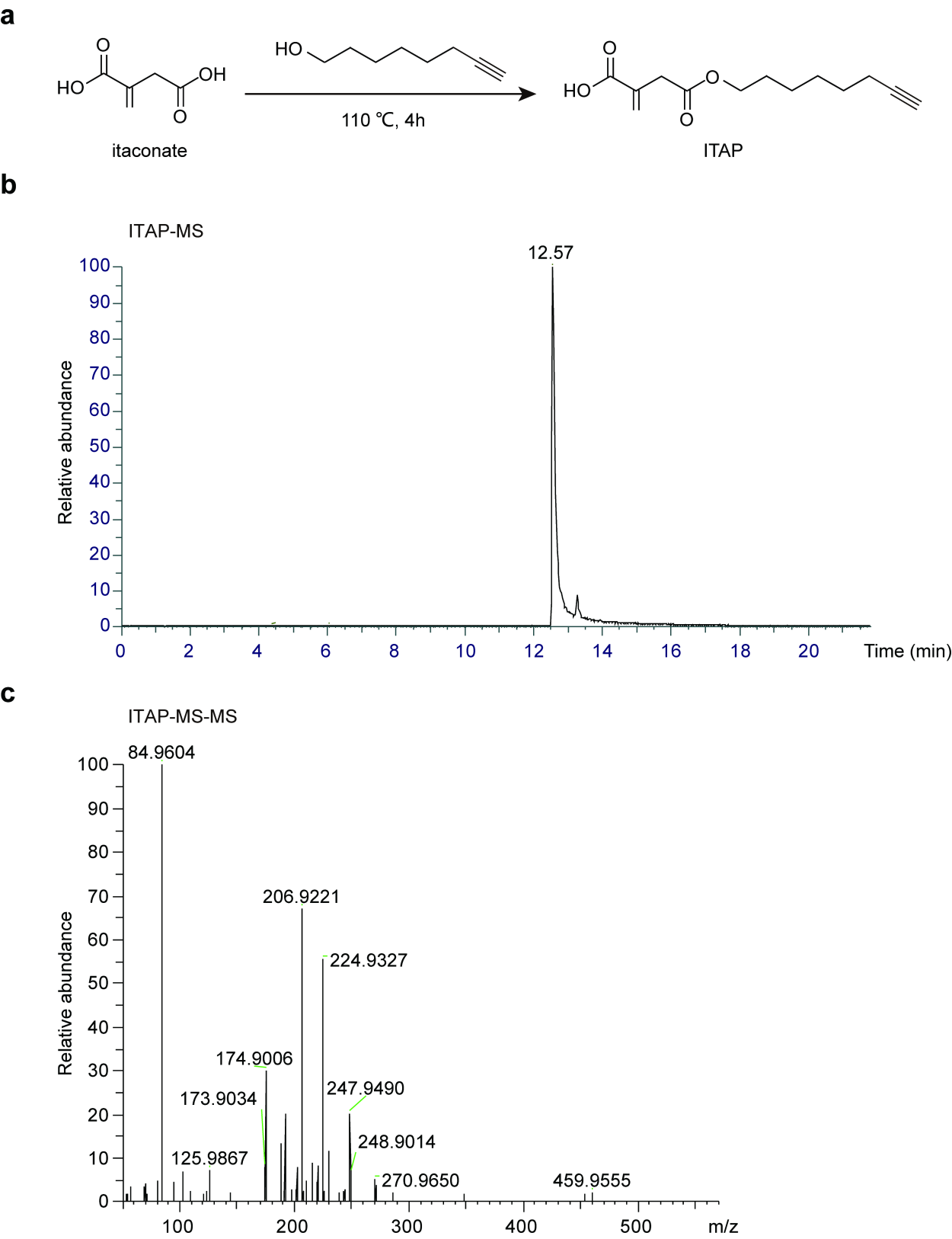
**

**Supplementary Figure S1 (a)** Synthesis scheme of (itaconate–alkyne probe) ITAP. Design of ITAP for capturing the itaconate targets. **(b, c)** ITAP identification with UHPLC-MS method.

**
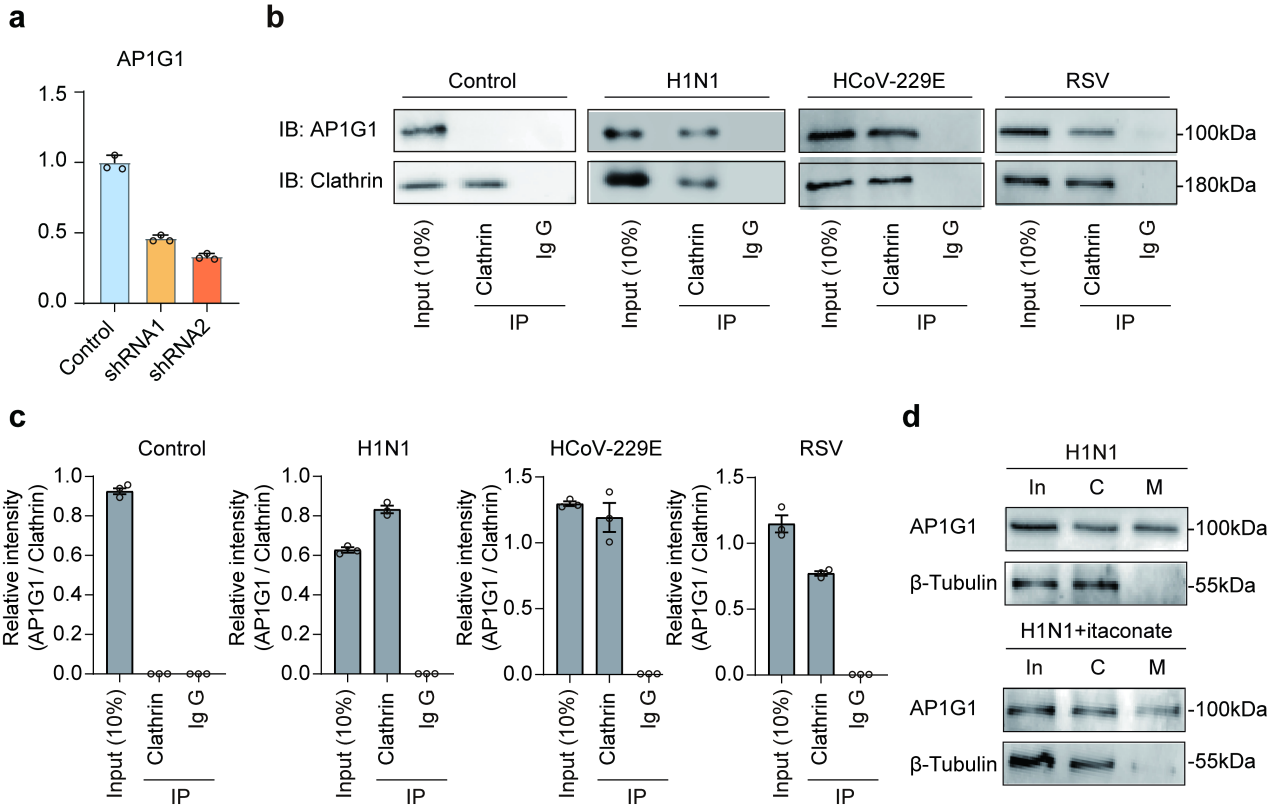
**

**Supplementary Figure S2 (a)** AP1G1 knock-down efficiency in BEAS-2B cells. The data represents three replicates. **(b)** CO-IP experiments show that AP1G1 did not interact with clathrin under normal conditions. However, AP1G1 exhibited interaction with clathrin during three different viral invasions: H1N1, HCoV-229E, and RSV. **(c)** Quantitative statistics of CO-IP experiments. Data are presented as the mean ± SEM from three independent experiments. **(d)** Western blot assay showing the subcellular localization of AP1G1 upon itaconate treatment, n = 3. In = input, C = cytoplasm, M = cell membrane.

**
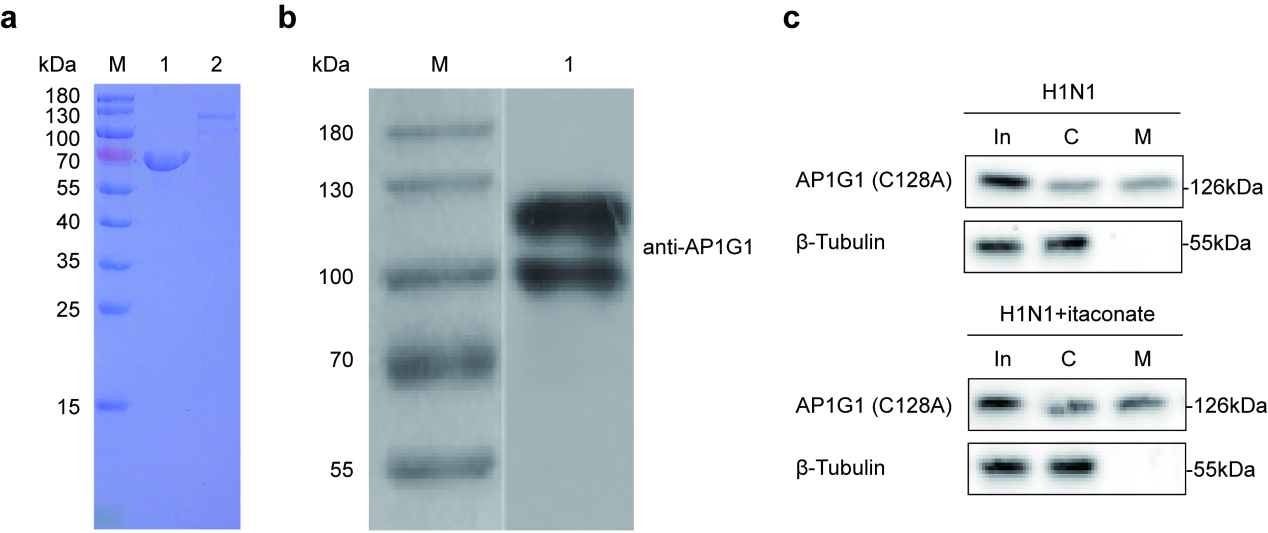
**

**Supplementary Figure S3 (a)** GST**-**AP1G1 recombinant protein identification with Coomassie Brilliant Blue, lane M = Marker, 1 = BSA, 2 = GST-AP1G1. **(b)** GST**-**AP1G1 recombinant protein identification with immunoblotting method, lane M = Marker, 1 = GST-AP1G1. **(c)** Subcellular localization of AP1G1 in C128A BEAS-2B cells, n = 3.

**
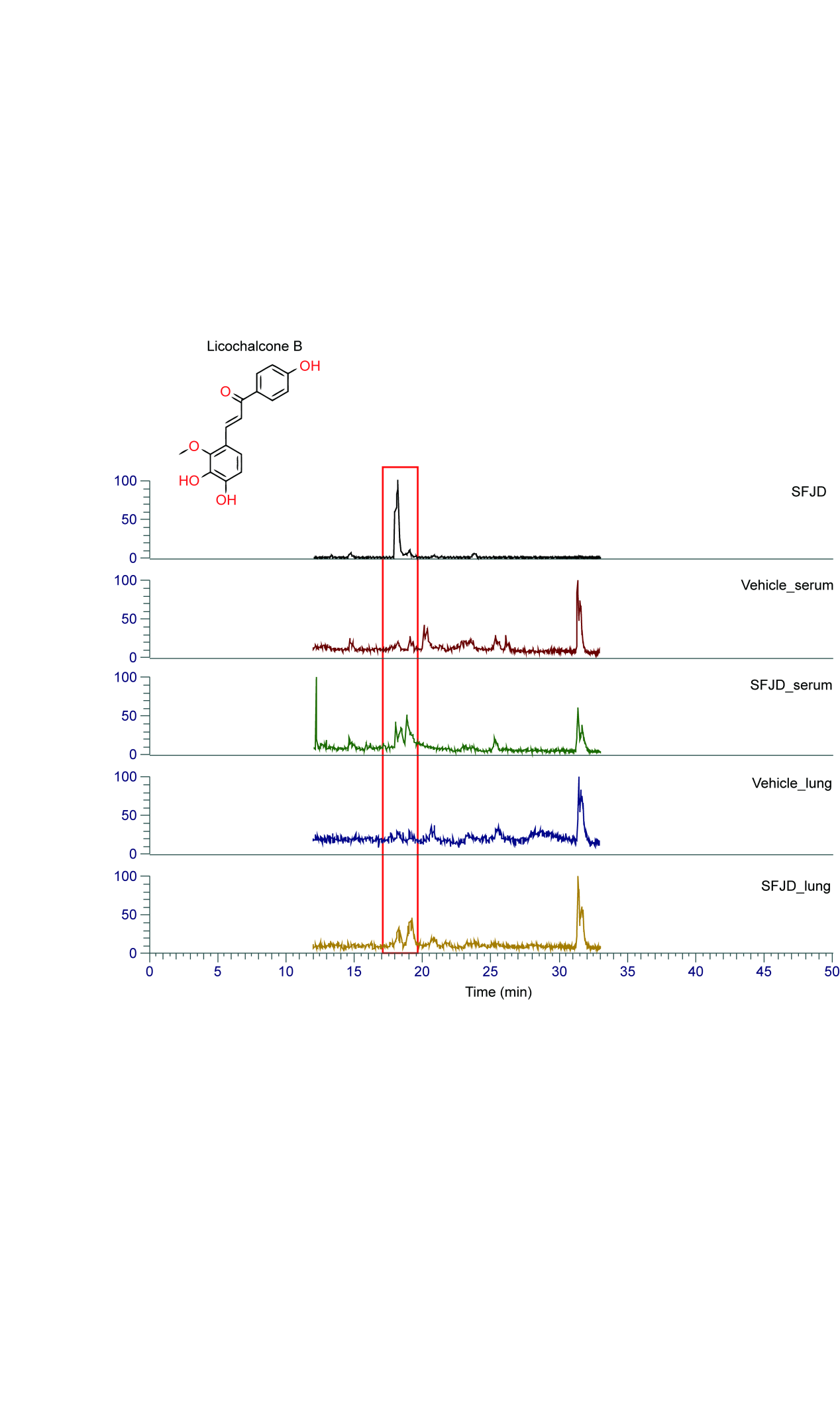
**

**Supplementary Figure S4** UHPLC-MS result demonstrating distribution and relative abundance of Licochalcone B in Licochalcone B extraction, serum, and lung tissue of mice. The data represents three replicates.

1. **REFERENCES**

[1] Chen J,Zhang X,Cao R,Lu X,Zhao S,Fekete A, et al. Serum 27-nor-5beta-cholestane-3,7,12,24,25 pentol glucuronide discovered by metabolomics as potential diagnostic biomarker for epithelium ovarian cancer. J Proteome Res. 2011;10(5):2625-32. <https://doi.org/10.1021/pr200173q>.

[2] Naz S,Vallejo M,Garcia A,Barbas C. Method validation strategies involved in non-targeted metabolomics. J Chromatogr A. 2014;135399-105. <https://doi.org/10.1016/j.chroma.2014.04.071>.

[3] Zhong HA,Almahmoud S. Docking and Selectivity Studies of Covalently Bound Janus Kinase 3 Inhibitors. Int J Mol Sci. 2023;24(7):6023. <https://doi.org/10.3390/ijms24076023>.

[4] Goodsell DS,Autin L,Olson AJ. Illustrate: Software for Biomolecular Illustration. Structure. 2019;27(11):1716-20.e1. <https://doi.org/10.1016/j.str.2019.08.011>.
